# Clinical improvement after change of therapy from tafamidis to patisiran in progressive TTR amyloidosis post-liver transplantation

**DOI:** 10.1007/s00415-022-10978-3

**Published:** 2022-02-06

**Authors:** Catherine Bulinski, Thomas Discher, Wiebke Rutsatz, Birgit Assmus, Heidrun H. Krämer

**Affiliations:** 1 Department of Internal Medicine, Infeciology, Justus Liebig University, Klinikstrasse 33, 35392 Giessen, Germany; 2 Department of Cardiology, Justus Liebig University, Giessen, Germany; 3 Department of Neurology, Justus Liebig University, Giessen, Germany

Dear Sirs,

Hereditary amyloidosis with autosomal-dominant mutations in the transthyretin-gene (TTR) represents a severe multi-system disorder. Treatment options are now available for TTR familial amyloid polyneuropathy (TTR-FAP): patisiran [1], inotersen [2], tafamidis [3] and orthotopic liver transplantation (OLT). It is known that TTR-cardiomyopathy and neurologic symptoms progress in many transplant recipients. This disease progression after transplantation occurs mainly in patients who are late stage with (p.Val50Met) or have a mutation other than (p.Val50Met) [4]. In these cases, the management strategy is unclear. One small study showed stable neuropathy scores during 12-month treatment with Inotersen after OLT [5]. Tafamidis stabilized TTR-FAP symptoms in one woman for 18 months [6]. Patisiran stabilizes neurological symptoms for 12 months after OLT [7]. No data are available, so far, on how to escalate amyloidosis therapy in case of disease progression after OLT during ongoing therapy with one of the approved agents.

We herewith present a case of a young man who had severe TTR-FAP progression after OLT. Tafamidis therapy did not stop disease progression, but the patient showed clinical improvement of the TTR-FAP after switch to patisiran.

A now 44-year-old man was diagnosed with TTR-FAP in 2012 caused by a rare TTR mutation (p.Glu54Gly) at the age of 36 years. The diagnosis occurred early because of his positive family history. Pre-existing conditions included chronic hepatitis B and D. At diagnosis, he reported autonomic disturbance causing gastrointestinal symptoms (constipation and diarrhoea) and noticed signs of small-fibre involvement (inability to detect temperatures) for approximately 1 year. Physical examination revealed slightly reduced muscle strength in the extensor muscles of the toes, distal hypesthesia in both feet, pallhypesthesia with 4/8 at the first metatarsophalangeal joints and absent achilles tendon reflexes. Nerve conduction studies (NCS) detected absent sensory responses in the lower limbs and decreased compound muscle action potential (CMAP) of the peroneal nerves. The mNIS + 7score was 31 points [1]. Echocardiography revealed an inhomogeneous echotexture of the myocardium, a slightly reduced systolic function with normal radial and impaired longitudinal function and increased septal thickness (16 mm). A cardiac MRI performed in 2013 confirmed reduced systolic function (ejection fraction 52%) and septal hypertrophy. Further, it showed a diffuse late gadolinium enhancement with a non-ischaemic pattern as described in cardiac involvement in amyloidosis. The patient was diagnosed with NYHA stadium I-II and sensorimotor axonal polyneuropathy (Coutinho stage 1). Oral therapy with tafamidis was started. A living split-liver transplantation was performed 2013 without any complications. After OLT, tafamidis medication was discontinued. However, the symptoms of the polyneuropathy slowly increased. In spring 2018, tafamidis medication was re-established. By this time, the patient’s complaints comprised of weakness in the legs, sensory loss in the feet, paraesthesia in the hands and postprandial hypotonia indicating autonomic neuropathy. Physical examination revealed paresis of the small hand muscles (MRC 4/5), the extensor muscles of the feet (MRC 2/5), areflexia at the lower extremities and pallanaesthesia at the first metatarsophalangeal joints. NCS detected absent sensory responses in all limbs and absent CMAP of the peroneal and significantly reduced CMAP of the tibial nerves (mNIS-7 score: 149.5). Echocardiography showed rather constant hypertrophy of the left ventricular septum with thickness of 17 mm and stable ejection fraction of 55%, but no diastolic dysfunction. However, the symptoms further advanced until the patient was unable to walk stairs and required permanent assistance while walking due to increasing gait unsteadiness (Coutinho stage 2). At the beginning of 2019, treatment with patisiran was started. At this time, physical examination revealed additional paresis of all small hand muscles (MRC 3/5), flexor muscles of the feet (MRC 4/5) and toes (MRC 3/5) and of the proximal leg muscles (MRC 4/5), areflexia and hypesthesia on the lower arms and legs. NCS showed now absent CMAP in the lower legs and significantly reduced CMAP in the upper limbs. Nerve sonography detected enlarged cross sectional areas of the median nerves and ulnar nerves at the upper arm (mNIS-7 score: 183.5). The patient’s TTR-FAP symptoms slowly improved during patisiran therapy. After 18 months of treatment, he could walk independently with two dynamic peroneal splints and climb 7–8 stairs with assistance (mNIS-7 score: 172.5). In addition, echocardiography in 2020 revealed a preserved ejection fraction, and no evidence of diastolic dysfunction (normal E/e´, only slightly enlarged left atrium with 30.5 ml/m^2^). By cardiac MRI, a septal hypertrophy of 20 mm was detected, as well as an increased amount of late gadolinium enhancement. Native T1 and T2 relaxation time were elevated. Thus, despite preserved left ventricular function, a progressive cardiac involvement, as suggested by increasing septal thickness, cannot be ruled out. However, as a major limitation, echocardiography and MRI were used across different centres, and detailed off-line core-lab analysis was not available. NCS remained stable compared to the investigation in 2019 (Table 1).

**Table 1:** Depicts clinical data of our patient over the observation period

	2012	2018	2019	2020
Clinical examination				
Coutinho stage	1	2	2	2
mNIS7 score	31	149.5	183.5	172.5
Nerve conduction studies				
Medial nerve, sensory	SNAP: 8 µV NCV: 52 m/s	Absent	Absent	Absent
Medial nerve, motor	CMAP: 11 mV NCV: 54 m/s	CMAP: 4.8 mV NCV: 48 m/s	CMAP: 0.9 mV NCV: 44 m/s	CMAP: 0.6 mV NCV: 46 m/s
Sural nerve	Absent	Absent	Absent	Absent
Peroneal nerve	CMAP: 2.5 mV NCV: 41 m/s	Absent	Absent	Absent
Tibial nerve	CMAP: 8.0 mV NCV: 43 m/s	CMAP: 0.9 mV NCV: 38 m/s	Absent	Absent
Cardiological examinations				
NYHA stadium	I–II	I–II	I–II	I–II
Septal thickness	16 mm	17 mm	18 mm	20 mm
Ejection fraction	52%	55%	55%	71%

*SNAP* sensory nerve action potential, *NCV* nerve conduction velocity, *CMAP* compound muscle action potential

This case provides evidence that change of treatment regimen in patients with disease progression after OLT is a valuable option. One possible explanation for the different therapeutic effect is the different mode of action of tafamidis and patisiran. Whereas tafamidis [3] acts as a TTR-stabilator, patisiran inhibits hepatic synthesis of transthyretin [1]. It is known that amyloidosis associated symptoms after OLT progress due to deposition of healthy amyloid on old TTR-amyloid deposits [4]. Since patisiran leads to significant diminished serum TTR levels even after OLT [7], this mechanism might be reduced.

The cardiac amyloidosis showed no change in our study. However, cardiac examinations were performed at multiple centres with different examination protocols, which limits comparability and interpretability of the results. Most importantly, no MRI-derived quantitative baseline septal thickness (2013) was available. However, a dramatic deterioration of cardiac function can be excluded. Although patisiran is not approved in the cardiac indication, previous studies demonstrated positive effects of patisiran on cardiac parameters, although results were very heterogeneous with respect to reductions in MRI-derived extracellular volumes as an indicator of cardiac TRR-amyloid regression of [8]. However, analysis of cardiac parameters in the APOLLO study showed, among other parameters, a reduction of mean left ventricular wall thickness and improved global longitudinal strain [9].

The number of TTR-amyloidosis patients post-OLT will further decrease over the next years. However, in these patients, gene-silencing therapy might be an option. Although the use of patisiran improved the clinical status of the patient with the rare mutation (p.Glu54Gly), it cannot be concluded that this positive effect will also occur in patients with other TTR mutations. In summary, larger studies are needed to establish evidence-based treatment recommendations for medical therapy of TTR-amyloidosis patients after OLT.

## References

[1] Adams D, Gonzalez-Duarte A, O'Riordan WD (2018). Patisiran, an RNAi therapeutic, for hereditary transthyretin amyloidosis. N Engl J Med.

[2] Benson MD, Waddington-Cruz M, Berk JL (2018). Inotersen treatment for patients with hereditary transthyretin amyloidosis. N Engl J Med.

[3] Coelho T, Maia LF, Martins da Silva A (2012). Tafamidis for transthyretin familial amyloid polyneuropathy: a randomized, controlled trial. Neurology.

[4] Ericzon B-G, Wilczek HE, Larsson M (2015). Liver transplantation for hereditary transthyretin amyloidosis: after 20 years still the best therapeutic alternative?. Transplantation.

[5] Moshe-Lilie O, Dimitrova D, Heitner SB (2020). TTR gene silencing therapy in post liver transplant hereditary ATTR amyloidosis patients. Amyloid.

[6] Romero-Imbroda J, Sagrario-Fustero T, Del Canto-Pérez C (2017). Tafamidis for a transplant patient with transthyretin amyloid polyneuropathy. J Clin Neurol.

[7] Muñoz-Beamud F, Coelho T, Gillmore JD (2021). 2021 peripheral nerve society virtual event. J Peripher Nerv Syst.

[8] Fontana M, Martinez-Naharro A, Chacko L (2021). Reduction in CMR derived extracellular volume with patisiran indicates cardiac amyloid regression. JACC Cardiovasc Imaging.

[9] Solomon SD, Adams D, Kristen A (2019). Effects of patisiran, an RNA interference therapeutic, on cardiac parameters in patients with hereditary transthyretin-mediated amyloidosis. Circulation.

